# The Impact of Higher Than Recommended Gestational Weight Gain on Fetal Growth and Perinatal Risk Factors—The IOM Criteria Reconsidered

**DOI:** 10.3390/ijerph21020147

**Published:** 2024-01-29

**Authors:** Sylvia Kirchengast, Josef Fellner, Julia Haury, Magdalena Kraus, Antonia Stadler, Teresa Schöllauf, Beda Hartmann

**Affiliations:** 1 Department of Evolutionary Anthropology, University of Vienna, 1030 Vienna, Austriaa11832972@unet.univie.ac.at (J.H.); a11832783@unet.univie.ac.at (M.K.);; 2 Department of Gynecology and Obstetrics, Clinic Donaustadt, 1030 Vienna, Austria; beda.hartmann@gesundheitsverbund.at

**Keywords:** excessive gestational weight gain, intrauterine growth, newborn size, pregnancy outcomes, emergency caesarean section, perinatal risks

## Abstract

A too-high gestational weight gain, in combination with steadily increasing obesity rates among women of reproductive age, represents an enormous obstetrical problem, as obesity and high gestational weight gain are associated with enhanced fetal growth, low vital parameters, and increased cesarean section rates. This medical record-based study investigates the association patterns between too-low as well as too-high gestational weight gain, according to the 2009 Institute of Medicine (IOM) guidelines, and fetal growth, as well as birth mode and pregnancy outcome. The data of 11,755 singleton births that had taken place between 2010 and 2020 at the Public Clinic Donaustadt in Vienna, Austria, were analyzed. Birth weight, birth length, head circumference, APGAR scores, and pH values of the arterial umbilical cord blood described fetal growth as well as the vital parameters after birth. Gestational weight gain was classified as too low, recommended, or too high according to the different weight status categories of the IOM guidelines. Birth weight, birth length, and head circumference of the newborn were significantly increased (*p* < 0.001) among underweight, normal-weight, and overweight women who gained more weight than recommended. Among obese women, only birthweight was significantly (*p* < 0.001) higher among women who gained more weight than recommended. Furthermore, a too-high gestational weight gain was significantly associated with an increased risk of macrosomia and emergency cesarean sections among underweight, normal-weight, and overweight women, but not among obese ones. Obese and morbidly obese women experiencing excessive gestational weight gain showed no significantly increased risk of macrosomia or emergency cesarean section. However, among obese mothers, a too-low gestational weight gain reduced the risk of emergency cesarean sections significantly (*p* = 0.010). Consequently, the IOM recommendations for gestational weight gain fit only partly for pregnant women in Austria. In the case of obese and morbidly obese women, new guidelines for optimal pregnancy weight gain should be considered.

## 1. Introduction

The prevalence of overweight and obesity has risen dramatically in recent decades. Since 1975, the number of overweight and obese people worldwide has roughly doubled [1]. Therefore, today, around one-third of the world’s population is considered overweight or obese, and a further increase in prevalence is to be expected [2,3]. This trend is extremely worrying, as obesity is not only classified as a chronic disease but also represents a significant risk factor for numerous other non-communicable diseases [4,5]. A special problem is the increasing prevalence of obesity and overweight among women of reproductive age, a trend that can be observed worldwide [6,7,8]. The proportion of overweight and obese pregnant women is between 7 and 25% [9].

Women of reproductive age are particularly vulnerable, as overweight and obesity are risk factors both before and during pregnancy [10,11]. Obesity can prevent successful fertilization [12,13] on the one hand and negatively affect the course of a pregnancy, fetal growth, and delivery on the other [14,15,16,17]. Obese pregnant women are more likely to suffer from gestational diabetes, high blood pressure, and pre-eclampsia. Furthermore, they have an increased risk of premature birth and cesarean sections [18,19,20]. According to Devlieger et al. [9], the risk of an emergency cesarean section in obese women is 16.1%, compared to 6.6% in normal-weight mothers. The probability of giving birth to a large-for-gestational-age (LGA) child is also increased in overweight and obese women. The problem here is that LGA children have a higher risk of being overweight or obese in early childhood. In contrast, the risk of giving birth to a small-for-gestational-age (SGA) child decreases [9,19].

However, it is not only excessive initial weight that poses a problem; inadequate weight gain during pregnancy exacerbates the effect of prepregnancy obesity in overweight and obese women [21]. During pregnancy, there are changes in the woman’s body composition and weight gain, which is referred to as gestational weight gain (GWG) [22,23]. The physiological GWG is caused by the weight of the growing fetus and the increase in the mother’s body fluids and fat [22,23]. Weight gain during pregnancy is necessary to enable normal fetal development. However, studies show that too much or too little GWG can have negative effects on the vital parameters of the fetus and the newborn [23,24]. Excessive GWG is positively associated with LGA infants and macrosomia [18,23,25]. For this reason, the American Institute of Medicine (IOM) published the so-called IOM guidelines in 2009, which define optimal weight gain during pregnancy for the different maternal weight status categories and thus aim to reduce the risks associated with increased GWG [26,27]. The different categories are shown in Table 1 [27]. These recommendations for optimal GWG were originally intended for women living in the USA only and were therefore considered not applicable worldwide. For this reason, the IOM guidelines were primarily used in high-income Western countries [26]; however, the IOM guidelines are also used in middle- and low-income countries [25,28,29].

Despite this use of the IOM guidelines nearly worldwide, the results of some studies contradict the recommendations of the IOM guidelines: According to Kominiarek et al. [30], a weight reduction in obese pregnant women significantly reduced the risk of giving birth to LGA infants or requiring an emergency cesarean section. This contrasts with the 5 to 9 kg GWG recommended by the IOM that a pregnant woman with a BMI > 30 kg/m² should gain. In contrast, Faucher et al. [19] observed a lower risk of SGA but also of LGA newborns with an optimal GWG of 5 to 9 kg for obesity class I (BMI 30–34.9), 1 to less than 5 kg for obesity class II (35–39.9), and no GWG for obesity class III (BMI ≥ 40). The study by Devlieger et al. [9] also makes different GWG recommendations for obese women than the IOM: To minimize the risk of giving birth to LGA or SGA newborns, obese women in class I (BMI 30–34.9) should gain 0 kg. Class II obese women (35–39.9) are recommended to lose 4 kg, and pregnant women in obesity class III (BMI ≥ 40) are recommended to lose 5 kg. Consequently, there is still no consensus regarding the recommendations for optimal weight gain during pregnancy in overweight or obese pregnant women, although the higher risk of adverse pregnancy outcomes among obese women experiencing excessive gestational weight gain was confirmed in several recent studies [25,28,29]. The IOM guidelines are also used in Austria, a country with particularly sophisticated care for pregnant women. In Austria, all residents have social insurance that enables free medical treatment. For pregnant women, the so-called mother–child pass was developed in the 1970s to make pregnancy and birth safer and to ensure optimal care for mother and child. At least three prenatal examinations and eight postnatal examinations up to the child’s fourth year of life are mandatory. All examinations are free of charge. The recommendations for weight gain during pregnancy correspond to the IOM guidelines. However, the IOM guidelines have not yet been evaluated for pregnant Austrian women. Therefore, in the present study, the IOM guidelines for pregnant women and their effects on pregnancy outcomes will be examined in Austria. The following hypotheses are tested in detail:

(1) Overweight and obese women who experienced a gestational weight gain above the IOM guidelines have a higher risk of giving birth to macrosomic newborns and reduced neonatal APGAR scores.
(2) Overweight and obese women who experienced a gestational weight gain above the recommended IOM guidelines have a higher risk of needing an emergency cesarean section.

## 2. Participants and Methods

### 2.1. Data Set and Study Design

In this retrospective medical record-based study, data from 11,755 individual births that took place at the Donaustadt Clinic in Vienna between 2010 and 2020 were analyzed. The Donaustadt Clinic is one of the largest open maternity clinics in Vienna. Strict exclusion criteria were defined to obtain as homogeneous a sample as possible and to be able to analyze the associations between gestational weight gain and fetal growth, as well as the birth process and pregnancy outcome, without confounding factors. Therefore, the following exclusion criteria were defined: stillbirths, miscarriages, multiple births, premature births before the 37th gestational week, the use of artificial reproductive technology (ART), and congenital anomalies. Furthermore, coincident medical diseases such as diabetes mellitus or nephropathy and drug or alcohol abuse by the mother were strict exclusion criteria. On the other hand, the following inclusion criteria had to be fulfilled to be included in the analysis: all prenatal check-ups of the Austrian mother–child passport were performed; term birth (≥37th gestational week); spontaneous conception; a healthy newborn without congenital anomalies; no registered maternal diseases before and during pregnancy; and no pregnancy-related immunization. Between 1 January 2010 and 31 December 2020, 24,220 children were born in this hospital. Excluded from the study were 81 triplets and 1558 twins, 142 stillbirths, 2065 premature births, and 1222 newborns conceived using artificial reproduction techniques. A total of 7407 cases were excluded due to illnesses of the mother or the child and/or incomplete data sets. Finally, 11,755 data sets were included in the analysis. The study design was retrospective and based on medical records. Therefore, it was not possible to record parameters that were not documented in the standard medical history. Consequently, no information on the socioeconomic status, dietary patterns, and physical activity of the mother before and during pregnancy is available. However, as the Danube Clinic is a public hospital, it can be assumed that the mothers belong to Vienna’s middle class. All the mothers lived in Vienna.

The present study is part of a huge project focusing on the impact of environmental factors on fetal growth and obstetrical risk factors. For more detailed information, see Kirchengast and Hartmann [15,31,32] and Syböck et al. [17]. In line with the whole project, the study was conducted according to the guidelines of the Declaration of Helsinki and approved by the Ethics Committee of Vienna (responsible for public hospitals) (Protocol number: EK 19-274-VK 18 March 2020).

### 2.2. Maternal Parameters

The following maternal parameters were documented: maternal age in years, body weight before pregnancy and before birth, and body height. At the first examination during pregnancy (usually in the 8th week of gestation), the prepregnancy body weight was asked in the sense of a retrospective survey, and the current body weight was measured using a standard scale. The mean of the two values was calculated and interpreted as the prepregnancy weight. During the same examination, body height was measured by a trained person using an anthropometer. Before birth, the body weight was measured at the end of the pregnancy. The weight gain during pregnancy was calculated as the difference between the initial weight and the body weight before birth. Prepregnancy weight status was determined using the body mass index (BMI) (kg/m^2^). The following BMI classes were defined following WHO guidelines [33]:
≤18.49 kg/m² underweight 18.50–24.99 kg/m² normal-weight 25.00–29.99 kg/m² overweight30.00–39.99 kg/m² obese (grade 1 + 2)≥40.00 kg/m²morbidly obese (grade 3)

The weight gain was categorized separately for each BMI class according to the IOM guidelines into too low, according to the recommendations, and too high.

### 2.3. Newborn Parameters

Immediately after birth, the following parameters were documented for each newborn by a midwife experienced in measurement techniques: Birth weight (in g) was measured using a digital infant scale, birth length (in cm) using a standard measurement board for infants, and head circumference (in cm) using a standard tape. The birth weight of the newborns was measured in g using a newborn scale and categorized accordingly into four weight categories. The categories are made up as follows: <1500 g (very low birth weight), 1500–2499 g (low birthweight), 2500–3999 g (normal weight), and >4000 g (macrosomic) [34]. APGAR scores were recorded 1, 5, and 10 min after birth [35]. The pH value of the arterial umbilical cord blood, which represents an accurate, reproducible, and objective evaluation of oxygen deficiency during birth, was measured to one decimal place. Using the pH value of the umbilical cord blood is therefore recommended to evaluate the newborn outcome [36,37].

### 2.4. Obstetrical Parameters

The mother’s reproductive history, the number of previous pregnancies, miscarriages, and births were documented in the medical anamnesis. The birth position of the child was recorded (cephalic, breech, transverse, or other presentation). It was recorded whether labor was induced with medication. Another parameter recorded was the mode of birth, whereby a distinction was made between a spontaneous vaginal birth, a vacuum extraction, a planned cesarean section, and an emergency cesarean section.

### 2.5. Statistical Analysis

The statistical analysis was performed using the statistical package IBM SPSS version 27. In the first step, descriptive statistics were computed. To test group differences in maternal and newborn parameters between too-low weight gain, recommended weight gain, and too-high weight gain, according to the IOM recommendations, ANOVAs were carried out for each maternal BMI class separately. Bonferroni post hoc tests are then conducted to allow pairwise comparisons between all groups. To evaluate the risk of low birth weight, macrosomia, emergency cesarean section, and acidosis, the odds ratios with 95% confidence intervals were calculated for too high or too low maternal weight gain, with recommended weight gain as the reference group. The associations between IOM groups and birth modes were tested by Pearson Chi^2^ tests. A multiple linear regression analysis was carried out to test the association patterns between recommended GWG and newborn size corrected by maternal age, maternal body height, prepregnancy BMI, and nicotine consumption during pregnancy. Since the recommended GWG was coded into 3 categories, dummy variables of GWG were created, making multiple linear regression possible. The recommended GWG was kept as the reference category. The significance level is set to *p* < 0.05.

## 3. Results

### 3.1. Sample Description

Table 2 demonstrates the sample characteristics of mothers, newborns, and obstetrical parameters. The average maternal age was 30.3 years, with the youngest mother being 14 years old and the oldest mother 55 years old. The majority of mothers (61.7%) met the definition of normal weight. More than 20% of the women were overweight, and more than 11% were obese. Only 30.7% of mothers experienced weight gain that met the IOM’s recommendations for their weight status, while 47.1% of women experienced excessive weight gain during pregnancy. A total of 45.2% of the women were first-time mothers. More than 85% of the newborns were of normal weight; almost 12% weighed more than 4000 g and were therefore macrosomic. Less than 2% weighed less than 2500 g. The cesarean section rate was 14%, with emergency cesareans predominating.

### 3.2. IOM Recommendations According to Maternal Weight Status

**Figure 1:**
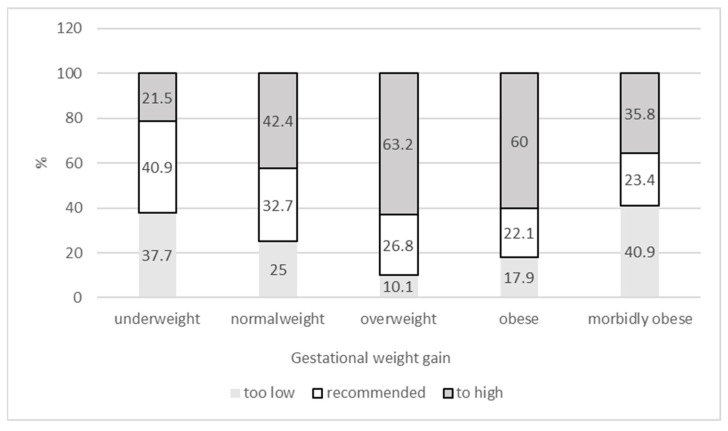
Gestational weight gain categories (IOM guidelines) according to weight status.

As presented in Table 2, only 30.7% of the mothers gained weight during gestation as recommended by the IOM, but more than 47% experienced a too-high GWG. Figure 1 presents the GWG categories according to prepregnancy weight status. As shown, the GWG categories differed highly significantly (<0.001) between the weight status categories. While more than 40% of underweight women showed a recommended GWG and only 21.5% of underweight women experienced a too-high GWG, more than 63% of overweight women gained too much weight, and only 26.8% of overweight women showed a GWG corresponding to the IOM recommendation. A similar pattern was found for women in obese grades 1 and 2. Among morbidly obese women, however, more than 40% gained less than the recommended weight, and only 35.8% experienced a too-high GWG.

### 3.3. Newborn Size and Vital Parameters According to IOM Recommendations

The comparison of newborn parameters between too-low, recommended, and too-high gestational weight gain for each respective weight status category is presented in Table 3. Gaining less than the recommended weight resulted in lighter and shorter newborns but also in fewer macrosomic ones, showing higher APGAR and umbilical arterial pH values, which indicate better vital parameters after birth. This was true of all maternal weight status groups. Also, for all maternal weight status groups, excessive gestational weight gain leads to significantly larger newborns. The APGAR scores, on the other hand, are usually lowest with excessive gestational weight gain. This was especially true of overweight and obese grade I and II mothers. Concerning morbidly obese mothers, however, a higher than recommended GWG was significantly associated with the highest birthweight, but no significant associations between GWG and the APGAR scores were observed.

The results of the Duncan analyses were corroborated by those of the multiple linear regression analyses. The impact of a too-low or a too-high GWG on newborn size and vital parameters (APGAR scores) corrected by maternal age, maternal body height, prepregnancy BMI, and nicotine consumption was tested. As presented in Table 4, a lower than recommended GWG was significantly associated with lower birth weight, shorter birth length, and a smaller head circumference of the newborns, independently of maternal age, maternal height, prepregnancy BMI, and nicotine consumption during pregnancy. In contrast, a higher than recommended GWG was significantly positively associated with increased newborn size, independent of the maternal parameters mentioned above.

### 3.4. Obstetrical Risk Factors According to IOM Recommendations

Odds ratios were computed to test the risk of a too-high or too-low GWG on obstetrical risk factors. The recommended weight gain was used as the reference category. As demonstrated in Table 5, a lower than recommended GWG increased the risk of giving birth to a low birthweight newborn but decreased the risk of giving birth to a macrosomic newborn, experiencing acidosis, or needing an emergency cesarean section. This was true of all prepregnancy weight status categories, except morbidly obese mothers. In this case, the risk of acidosis (OR 3.05) was significantly higher (*p* = 0.049) among mothers with a GWG lower than recommended.

A higher than recommended GWG increased the risk of giving birth to a macrosomic newborn, acidosis, and experiencing an emergency cesarean section significantly among normal-weight and overweight mothers. Among underweight mothers, a higher than recommended GWG was significantly associated with an increased risk of giving birth to a macrosomic newborn but with a significantly decreased risk of needing an emergency cesarean section (see Table 5). Concerning women classified as obese grades I or II, only an insignificantly increased risk of needing an emergency cesarean section (OR 1.19) and giving birth to a macrosomic newborn (OR 1.32) was observed. The risk of acidosis (OR 1.41), however, was significantly increased among obese women experiencing a GWG higher than recommended. Among morbidly obese women, neither a significantly increased risk of emergency cesarean section nor an increased risk of giving birth to macrosomic newborns or acidosis were observed. On the other hand, a lower than recommended GWG reduced the risk of giving birth to a macrosomic newborn significantly (*p* = 0.041).

## 4. Discussion

In 2009, the IOM published recommendations on optimal weight gain during pregnancy for the individual weight status categories [26,27]. Based on these recommendations, the present study analyzed the significance of gestational weight gain for fetal growth and obstetric risks in an Austrian sample. Particular attention was paid to overweight and obese pregnant women. Two hypotheses were tested. First, it was predicted that overweight and obese pregnant women who had a too-high GWG, according to the IOM recommendations, showed an increased risk of giving birth to macrosomic newborns and reduced APGAR scores. Second, the hypothesis was tested that overweight and obese pregnant women who experienced weight gain above the IOM recommendations had an increased risk of emergency cesarean section.

Both hypotheses could be partially verified. In all maternal weight status classes, a higher than recommended gestational weight gain led to a significantly higher birth weight. Macrosomia, which is caused by different factors, is a special problem that can be discussed in relation to excessive GWG, mainly because this risk factor can be favorably influenced by modifying behavior during pregnancy [24]. In the present study, however, the risk of giving birth to a macrosomic child was only significantly increased in underweight, normal-weight, and overweight women who gained more weight than recommended. These findings follow those of various other studies that show a significant positive association between excessive weight gain and large-for-gestational-age or macrosomic newborns [24,25,28]. Obese and morbidly obese mothers, however, did not show a significantly increased risk of macrosomia; the risk was slightly higher but not significantly. This result thus contrasts with that of Hung and Hsieh [38], who were able to show that the risk of macrosomia is increased in overweight and obese women if they gain too much weight according to the IOM criteria. However, it should be noted that the proportion of morbidly obese mothers in this study was very low (1.2%). In overweight mothers, however, the risk of macrosomia was significantly increased if the weight gain was above the IOM recommendations. This corresponds to the findings of Langford et al. [39], who assumed that hyperglycemia might be the cause of increased fetal growth with excessive weight gain, as an increased insulin concentration is interpreted as growth-promoting for the fetus. However, the increased energy intake could also have a growth-promoting effect and thus favor macrosomia. Macrosomia represents an obstetrical risk factor because it could increase the risk of cesarean sections [39,40]. In the present study, a too-high GWG among obese and morbidly obese mothers did not increase the risk of an emergency cesarean section or the risk of macrosomic newborns. Among underweight, normal-weight, and overweight mothers who gained excessive weight during pregnancy, the risk of giving birth to a macrosomic newborn increased significantly. Among normal-weight and overweight women, the risk of needing an emergency cesarean section also increased significantly. In this case, an association between the increased risk of macrosomia and emergency cesarean sections can be assumed. Similar findings were reported by Champion and Harper [24].

The hypothesis that a higher than recommended gestational weight gain is associated with significantly lower APGAR scores was also verified for normal-weight, overweight, and grades 1 and 2 obese women in this study. This also corresponds to the results of Nohr et al. [41] for a Danish sample and the study by Golawski et al. [42] for a Polish sample. The morbidly obese mothers in our study who had gained more weight than recommended, on the other hand, showed lower APGAR scores after 1 and 5 min than women who had gained weight according to the recommendations, but no significant differences were found. This could again be due to the low number of morbidly obese mothers and the fact that the number of morbidly obese women who gained excessive weight was especially small: more than 40% of morbidly obese women gained less than the recommended weight and only 35% gained more than the recommended weight. Another possible interpretation would be that the IOM guidelines for obese and morbidly obese women specify too low a threshold for excess GWG, and therefore no increased perinatal risks can yet be observed.

Our study partially confirmed the hypothesis that overweight and obese women who have a GWG above the recommended IOM guidelines are at higher risk of an emergency cesarean section. A significantly increased risk of emergency cesarean section was found in normal-weight and overweight mothers who had gained more weight than recommended. On the other hand, in obese and morbidly obese mothers, there was no evidence of an increased risk of emergency cesarean section if they gained more weight than recommended. This finding is in contradiction to those of the recently published study of Robilliard [43], who reported an increased risk of cesarean sections among grade I and grade II obese women. Chen et al. [44], however, reported similar results as in the present study, whereby there was also a significantly higher probability of cesarean sections in overweight women, even if the GWG was also above the recommended guidelines. In their study with 2107 participants, Haile et al. [45] also observed a higher probability of unplanned cesarean sections or emergency cesarean sections if the weight gain was too high compared to an adequate or too low weight gain. For overweight women, an increased likelihood of emergency cesarean sections can also be confirmed by a Norwegian study, while a similar but statistically insignificant correlation was observed for obese women [46]. An increased cesarean section rate with excessive GWG can also be confirmed by a study conducted in Taiwan [38]. A similar result was reported by Xiong et al. [47], although planned cesarean sections and emergency cesarean sections were not separated here. Durst et al. [48], on the other hand, were able to determine for obese pregnant women from the USA that excessive GWG is associated with an increased cesarean section rate. In the present study, a lower than recommended GWG, however, reduced the risk of emergency cesarean sections significantly among obese women. This observation corresponded to the findings of Sun et al. [49], who also reported a decreased risk of cesarean sections among women gaining less than the recommended weight during pregnancy. It is still not clear what mechanisms cause the associations between GWG and the risk of needing a cesarean section; however, a negative association between GWG and cervical dilatation during childbirth was reported [50]. The higher the GWG, the lower the cervical dilatation during the first stage of labor [50]. On the other hand, an excessive amount of abdominal fat may distort the birth canal and make the descent of the fetal head impossible [51].

The results of the present study show that the IOM recommendations for underweight, normal-weight, and overweight pregnant Austrian women are justified. For obese and morbidly obese pregnant women, however, there is no significantly increased risk of macrosomia or emergency cesarean sections if they gain too much weight, according to the IOM guidelines. This result is astonishing, since obesity and, above all, morbid obesity are independent risk factors for macrosomia and perinatal problems, as is excessive GWG. An additive effect of these two risk factors would therefore be expected. This could not be confirmed in the present study. The question therefore arises as to whether the IOM recommendations for obese pregnant women in Austria are appropriate. Further studies are necessary. The IOM definition of a low GWG should also be critically scrutinized. In the present study, too little weight gain according to IOM guidelines was significantly associated with a smaller newborn size, regardless of maternal co-factors such as smoking, age, body height, or body weight. Also, low weight gain in underweight and normal-weight women significantly increased the risk of a low birth weight below 2500 g; in overweight and grades 1 and 2 obese women, the risk was also increased but not significantly; and in morbidly obese women, the risk of giving birth to an underweight baby was even lower.

The meta-analysis by Kapadia et al. [52] also showed that the risk of macrosomia and cesarean section in newborns, as well as pre-eclampsia and gestational hypertension, was reduced in obese pregnant women if weight gain during pregnancy was below the recommended guidelines. However, no recommendation was made in this study for obese pregnant women to aim for weight gain below the recommended range according to IOM guidelines. There is evidence from the American College of Obstetrics and Gynecology that obese pregnant women with a lower weight gain than recommended and an appropriately developed fetus have a more favorable prognosis than those with a weight gain according to the guidelines [52]. According to Mustafa et al. [53], there is no significant evidence of a risk of SGA or preterm birth if obese mothers have a weight gain below the recommended guidelines during pregnancy. Consequently, the meaningfulness of the IOM guidelines must be questioned even based on the results of the sample of the present study, which are partly comparable with those of Dalfra et al. [23]. According to this study, the IOM recommendations for underweight and normal-weight women appear to be fundamentally suitable. This also corresponds to the results of the present study. In overweight and obese pregnant women with excessive weight gain, however, Dalfra et al. [23] often observed negative consequences for the newborn and the birth process. This contrasts with the results of the present study. The IOM definitions of excessive weight gain for obese and morbidly obese women showed no increased risks of macrosomia or emergency cesarean sections in our Austrian sample. Further research to clarify the causes of these findings is necessary.

## 5. Limitations

The present study has some limitations. The main limitation is the study’s design. A retrospective medical record-based study contains only the information that is documented as standard in the medical records. This means that behavioral parameters such as diet, physical activity, or information on socioeconomic status were not available. This limits the possibilities for interpreting the results because no correction for these confounding factors was possible. In addition, this is a single-center study, so it cannot be assumed that the results are generally valid. Another limitation is the low number of morbidly obese women (1.2%), although the sample of over 11,000 cases can be described as representative. The group sizes of the individual weight status classes, however, are very different. As expected, the group of pregnant women classified as normal-weight was the largest, with 7257 cases (61.7%). The other weight status groups were smaller. This makes a comparison difficult. Finally, we must mention that a longitudinal or prospective study including behavioral and socioeconomic data would have been methodologically more favorable. But in this case, it would not have been possible to obtain such a large sample.

## 6. Conclusions

In this study, the effects of excessive or insufficient weight gain during pregnancy on fetal growth and obstetrical problems were tested in an Austrian sample. The usefulness of the IOM guidelines was partially confirmed. Excessive weight gain is associated with enhanced fetal growth and lower APGAR scores, but also with increased risks of macrosomia, emergency cesarean section, and acidosis in normal-weight and overweight mothers. From a public health perspective, these findings are important, and the GWG of normal-weight and overweight pregnant women should be monitored carefully. Obese and especially morbidly obese women, however, did not show significantly increased risks with excessive weight gain. Consequently, in the future, special attention should be paid to adherence to the IOM guidelines in normal-weight and overweight women. Appropriate monitoring and individual advice in the sense of personalized medicine concerning dietary patterns, physical activity, and especially weight gain recommendations are of high priority. The IOM guidelines for obese and morbidly obese women, however, should be critically reviewed. Further studies are necessary.

## Figures and Tables

**Table 1:** Recommended gestational weight gain depending on prepregnancy BMI [27].

Prepregnancy BMI (in km/m²)	Recommended Gestational Weight Gain (in kg)
≤18.5 kg/m²	12.5–18 kg
18.8–24.9 kg/m²	11.5–16 kg
25.0–29.9 kg/m²	7.0–11.5 kg
≥30.0 kg/m²	5.05–9 kg

**Table 2:** Sample characteristics of maternal and newborn parameters.

		Mean (SD)	Range	n
**Maternal Parameters**				
Age (years)		30.3 (5.5)	14.0–55.0	11,755
Body height (cm)		165.4 (6.3)	140–191	11,755
Prepregnancy weight (kg)		65.8 (14.3)	36–160	11,755
End of pregnancy weight (kg)		80.1 (14.4)	43–170	11,755
Gestational weight gain (kg)		14.3 (5.8)	−17–42	11,755
GWG according to IOM recommendations				
	GWG too low			2600 (22.1%)
	Recommended GWG			3614 (30.7%)
	GWG too high			5541 (47.1%)
Prepregnancy BMI (kg/m^2^)		24.01 (4.89)	13.39–57.27	
<18.50 kg/m^2^				717 (6.1%)
18.50–24.99 kg/m^2^				7257 (61.7%)
25.00–29.99 kg/m^2^				2443 (20.8%)
30.00–39.99 kg/m^2^				1201 (10.2%)
≥40.00 kg/m^2^				137 (1.2%)
First-time mothers				5309 (45.2%)
Number of pregnancies		2.3 (1.4)	1–15	11,755
Number of births		1.8 (0.9)	1–10	11,755
Number miscarriages		0.4 (0.8)	0–14	11,755
Nicotine consumption during pregnancy				1593 (13.6%)
**Newborn parameters**				
Birth length (cm)		50.9 (2.1)	33.0–65.0–56.0	11,741
Head circumference (cm)		34.4 (1.5)	29.0–53.0	11,741
Birth weight (g)		3450.0 (457.2)	1700–5350	11,755
	<2500 g			187 (1.6%)
	2500–3999 g			10,176 (86.6%)
	≥4000 g			1392 (11.8%)
APGAR value 1 min		9.1 (1.0)	0–10	11,745
APGAR value 5 min		9.8 (0.7)	1–10	11,745
APGAR value 10 min		9.9 (0.3)	1–10	11,745
pH value (arterial cord blood)		7.3 (0.1)	6.5–7.6	11,672
Newborn sex				
	Male			6040 (51.4%)
	Female			5715 (48.6%)
**Obstetrical parameters**				
Birth induction				2541 (21.6%)
Birth mode				
	Spontaneous			9472 (80.6%)
	Vacuum extraction			640 (5.4%)
	Planned cesarean section			726 (6.2%)
	Emergency cesarean section			917 (7.8%)
Child presentation				
	Cephalic presentation			11,204 (95.6%)
	Breech presentation			496 (4.2%)
	Transverse presentation			18 (0.1%)
Acidosis ph-value < 7.2				2604 (22.3%)

**Table 3:** Newborn parameters according to gestational weight gain recommendations for each respective maternal prepregnancy weight category (Duncan analyses + Bonferroni).

Parameter		Gestational Weight Gain According to the IOM			
		Too Low	Recommended	Too High	
		Mean (SD)	Mean (SD)	Mean (SD)	*p*-Value
**Maternal BMI < 18.50 kg/m^2^**		n = 270	n = 293	n = 154	
	Birth weight (g)	3137.5 (401.2) ^bc^	3264.4 (396.7) ^ac^	3414.3 (436.3) ^ab^	<0.001
	Birth length (cm)	49.8 (1.9) ^bc^	50.3 (1.8) ^ac^	50.9 (1.9) ^ab^	<0.001
	Head circumference (cm)	33.7 (1.3) ^c^	34.0 (1.3) ^c^	34.3 (1.3) ^ab^	<0.001
	NA-pH-value	7.27 (0.08)	7.24 (0.09)	7.26 (0.08)	0.306
	APGAR 1 min	9.20 (1.02)	9.16 (0.93)	9.13 (0.85)	0.754
	APGAR 5 min	9.85 (0.56)	9.85 (0.66)	9.88 (0.43)	0.866
	APGAR 10 min	9.97 (0.23)	9.95 (0.40)	9.96 (0.25)	0.782
**Maternal BMI 18.50–24.99 kg/m^2^**		n = 1813	n = 2370	n = 3074	
	Birth weight (g)	3309.3 (419.5) ^bc^	3402.8 (416.4) ^ac^	3528.7 (447.4) ^ab^	<0.001
	Birth length (cm)	50.4 (1.9) ^bc^	50.8 (1.9) ^ac^	51.3 (2.1) ^ab^	<0.001
	Head circumference (cm)	34.1 (1.3) ^bc^	34.3 (1.4) ^ac^	34.5 (1.4) ^ab^	<0.001
	NA-pH-value	7.27 (0.08) ^bc^	7.26 (0.09) ^ac^	7.25 (0.08) ^ab^	<0.001
	APGAR 1 min	9.19 (0.94) ^c^	9.15 (0.89) ^c^	9.03 (1.08) ^ab^	<0.001
	APGAR 5 min	9.85 (0.56)	9.85 (0.58) ^c^	9.79 (0.69) ^b^	<0.001
	APGAR 10 min	9.96 (0.28)^c^	9.96 (0.31) ^c^	9.94 (0.35) ^ab^	0.028
**Maternal BMI 25.00–29.99 kg/m^2^**		n = 246	n = 654	n = 1543	
	Birth weight (g)	3310.9 (416.2) ^bc^	3479.4 (435.3) ^ac^	3584.3 (475.2) ^ab^	<0.001
	Birth length (cm)	50.4 (2.09) ^bc^	50.9 (1.9) ^ac^	51.4 (2.2) ^ab^	<0.001
	Head circumference (cm)	34.1 (1.5) ^bc^	34.4 (1.4) ^ac^	34.7 (1.6) ^ab^	<0.001
	NA-pH-value	7.28 (0.08) ^bc^	7.26 (0.09) ^ac^	7.25 (0.09) ^ab^	<0.001
	APGAR 1 min	9.21 (0.85)	9.07 (1.09)	9.04 (0.94)	0.037
	APGAR 5 min	9.88 (0.47)	9.79 (0.72)	9.78 (0.67)	0.091
	APGAR 10 min	9.93 (0.39)	9.93 (0.33)	9.94 (0.45)	0.993
**Maternal BMI 30.00–39.99 kg/m^2^**		n = 265	n = 215	n = 721	
	Birth weight (g)	3466.5 (465.9) ^bc^	3541.8 (500.1) ^ac^	3626.0 (498.8) ^ab^	<0.001
	Birth length (cm)	51.0 (1.9)	51.3 (1.9)	51.5 (2.1)	0.054
	Head circumference (cm)	34.5 (1.3) ^c^	34.7 (1.5)	34.7 (1.7)	0.098
	NA-pH-value	7.26 (0.09) ^c^	7.26 (0.09) ^c^	7.25 (0.09) ^ab^	0.014
	APGAR 1 min	9.10 (0.83) ^c^	9.04 (0.99) ^c^	8.85 (1.23) ^ab^	0.001
	APGAR 5 min	9.80 (0.72) ^c^	9.80 (0.63) ^c^	9.68 (0.84) ^ab^	0.024
	APGAR 10 min	9.96 (0.24)	9.91 (0.40)	9.92 (0.36)	0.349
**Maternal BMI ≥ 40.00 kg/m^2^**		n = 56	n = 32	n = 49	
	Birth weight (g)	3450.0 (468.5) ^c^	3547.5 (618.8)	3731.9 (475.5) ^a^	0.019
	Birth length (cm)	50.9 (2.0)	51.4 (2.3)	51.6 (1.9)	0.254
	Head circumference (cm)	34.5 (1.2)	34.6 (2.1)	35.0 (1.6)	0.199
	NA-pH-value	7.24 (0.08)	7.25 (0.07)	7.24 (0.08)	0.639
	APGAR 1 min	8.95 (0.92)	8.75 (1.32)	8.59 (1.31)	0.304
	APGAR 5 min	9.77 (0.47)	9.66 (0.87)	9.61 (0.86)	0.532
	APGAR 10 min	9.95 (0.23)	9.81 (0.64)	9.92 (0.34)	0.308

Legend: ^a^ = significantly different from too-low GWG; ^b^ = significantly different from recommended GWG; ^c^ = significantly different from too-high GWG.

**Table 4:** Associations between gestational weight gain categories and newborn size as well as vital parameters (APGAR scores). Multiple linear regression analyses.

		Coeff	*p*		95% CI	Coeff	*p*	95% CI
**Dependent Variable:**		**Birth Weight**				**APGAR 1 min**		
	Too-low GWG ^1^	−99.97	<0.001		−120.58–−79.17	0.06	0.011	0.01–0.11
	Too-high GWG ^1^	58.02	<0.001		48.76–67.28	−0.05	< 0.001	0.08–−0.03
	Maternal age	−0.29	0.762		−2.16–1.58	−0.01	0.365	−0.01–0.01
	Maternal body height	11.99	<0.001		10.37–13.61	0.01	< 0.001	0.01–0.02
	Prepregnancy BMI	14.55	<0.001		12.46–16.65	−0.01	< 0.001	−0.01–−0.00
	Smoking	−227.09	<0.001	−258.44–−195.74		0.05	0.145	−0.02–0.13
**Dependent Variable:**		**Birth length**				**APGAR 5 min**		
	Too-low GWG ^1^	−0.36	<0.001		−0.46–−0.27	0.02	0.270	−0.01–0.05
	Too-high GWG ^1^	0.22	<0.001		0.17–0.26	−0.03	< 0.001	−0.04–−0.01
	Maternal age	0.01	0.496		−0.01–0.01	0.02	0.741	−0.01–0.01
	Maternal body height	0.06	<0.001		0.05–0.06	0.01	0.061	0.01–0.01
	Prepregnancy BMI	0.05	<0.001		0.04–0.06	−0.01	0.007	−0.01–−0.00
	Smoking	−0.87	<0.001		−1.01–−0.72	0.03	0.169	−0.02–0.08
**Dependent Variable:**		**Head circumference**				**APGAR 10 min**		
	Too-low GWG ^1^	−0.17	<0.001		−0.24–−0.09	0.10	0.196	−0.01–0.03
	Too-high GWG ^1^	0.12	<0.001		0.09–0.15	−0.01	0.158	−0.01–0.01
	Maternal age	0.01	0.030		0.01–0.01	−0.01	0.244	−0.01–0.01
	Maternal body height	0.03	<0.001		0.03–0.04	0.01	0.617	−0.01–0.01
	Prepregancy BMI	0.03	<0.001		0.02–0.04	−0.01	0.011	−0.01–0.00
	Smoking	−0.55	<0.001		−0.66–−0.45	0.01	0.413	−0.01–0.03

Legend: GWG = gestational weight gain; ^1^ = reference category recommended GWG.

**Table 5:** Prevalence and odds ratios of emergency cesarean section, low birthweight, macrosomia, and acidosis according to GWG recommendations for each respective maternal prepregnancy weight category. Recommended GWG is the reference group.

Parameter		Gestational Weight Gains According to IOM							
		Recommended		Too Low			Too High		
		n (%)	n (%)		OR	*p*	n (%)	OR	*p*
**Maternal BMI < 18.50 kg/m^2^**									
	Emergency CS	19 (6.5%)	15 (5.6%)		0.85	n.s.	3 (1.9%)	0.29	0.035
	LBW > 2500 g	7 (2.4%)	12 (4.4%)		1.90	n.s.	3 (1.9%)	0.81	n.s.
	Macrosomia > 4000 g	10 (3.4%)	5 (1.9%)		0.53	n.s.	18 (11.7%)	3.75	0.001
	Acidosis pH < 7.20	69 (24.1%)	45 (16.7%)		0.63	0.029	32 (20.8%)	0.83	n.s.
**Maternal BMI 18.50–24.99 kg/m^2^**									
	Emergency CS	159 (6.7%)	107 (5.9%)		0.87	n.s.	267 (8.7%)	1.32	0.007
	LBW > 2500 g	36 (1.5%)	42 (2.3%)		1.54	0.049	37 (1.2%)	0.79	n.s.
	Macrosomia > 4000 g	191 (8.1%)	99 (5.5%)		0.66	0.001	458 (14.9%)	1.99	0.001
	Acidosis pH < 7.20	498 (21.2%)	336 (18.7%)		0.86	0.059	726 (23.8%)	1.16	0.022
**Maternal BMI 25.00–29.99 kg/m^2^**									
	Emergency CS	44 (6.7%)	13 (5.3%)		0.77	n.s.	156 (10.1%)	1.56	0.012
	LBW > 2500 g	9 (1.4%)	4 (1.6%)		1.19	n.s.	16 (1.0%)	0.75	n.s.
	Macrosomia > 4000 g	71 (10.9%)	12 (4.9%)		0.42	0.001	273 (17.7%)	1.77	0.001
	Acidosis pH < 7.20	136 (20.9%)	38 (15.9%)		0.71	n.s.	387 (25.2%)	1.26	0.031
**Maternal BMI 30.00–39.99 kg/m^2^**									
	Emergency CS	26 (9.8%)	8 (3.7%)		0.36	0.010	83 (11.88%)	1.19	n.s.
	LBW < 2500 g	4 (1.5%)	5 (2.3%)		1.55	n.s.	10 (1.4%)	0.92	n.s.
	Macrosomia > 4000 g	32 (17.5%)	30 (14.0%)		0.81	n.s.	153 (21.2%)	1.35	n.s.
	Acidosis pH < 7.20	56 (21.4%)	47 (22.0%)		1.04	n.s.	198 (27.6%)	1.41	0.047
**Maternal BMI ≥ 40.00 kg/m^2^**									
	Emergency CS	5 (15.6%)	4 (7.1%)		0.42	n.s.	83 (11.5%)	1.19	n.s.
	LBW < 2500 g	1 (3.1%)	1 (1.8%)		0.56	n.s.	0 (0.0%)	0.97	n.s.
	Macrosomia > 4000 g	8 (25.0%)	5 (8.9%)		0.29	0.041	15 (30.6%)	1.32	n.s.
	Acidosis pH < 7.20	4 (12.5%)	17 (30.4%)		3.05	0.049	14 (28.6%)	2.80	n.s.

Legend: LBW = low birth weight; CS = cesarean section; BMI = body mass index, n.s. = not significant.

## Data Availability

The datasets presented in this article are not readily available because., the data were obtained from the medical records of the Danube Hospital, Vienna, and are exclusively available with the permission of the Wiener Gesundheitsverbund.
